# Individual and combined effects of indoor home exposures and ambient PM_2.5_ during early life on childhood asthma in us birth cohort studies

**DOI:** 10.1097/EE9.0000000000000443

**Published:** 2025-12-23

**Authors:** Akihiro Shiroshita, Antonella Zanobetti, Brent A. Coull, Patrick H. Ryan, Soma Datta, Jeffrey Blossom, Emily Oken, James E. Gern, Heike Luttmann-Gibson, Eneida A. Mendonça, Sima K. Ramratnam, Sheryl L. Rifas-Shiman, Joanne E. Sordillo, Veronica A. Wang, Paloma I. Beamer, Daniel J. Jackson, Christine C. Johnson, Gurjit K. Khurana Hershey, Fernando D. Martinez, Rachel L. Miller, Katherine Rivera-Spoljaric, Edward M. Zoratti, Tina V. Hartert, Diane R. Gold, for the ECHO Cohort Consortium, P Brian Smith

**Affiliations:** a Division of Epidemiology, Department of Medicine, Vanderbilt University School of Medicine, Nashville, Tennessee; b Department of Environmental Health, Harvard T. H. Chan School of Public Health, Boston, Massachusetts; c Department of Biostatistics, Harvard T. H. Chan School of Public Health, Boston, Massachusetts; d Department of Pediatrics, University of Cincinnati, College of Medicine, Cincinnati, Ohio; e Division of Biostatistics and Epidemiology, Cincinnati Children’s Hospital Medical Center, Cincinnati, Ohio; f Channing Division of Network Medicine, Brigham and Women’s Hospital and Harvard Medical School, Boston, Massachusetts; g Center for Geographic Analysis, Harvard University, Cambridge, Massachusetts; h Department of Population Medicine, Harvard Medical School and Harvard Pilgrim Health Care Institute, Boston, Massachusetts; i Department of Pediatrics, University of Wisconsin School of Medicine and Public Health, Madison, Wisconsin; j Division of Biomedical Informatics, Cincinnati Children’s Hospital Medical Center and University of Cincinnati, Cincinnati, Ohio; k Asthma and Airways Disease Research Center, University of Arizona, Tucson, Arizona; l Department of Public Health Sciences, Henry Ford Health, Detroit, Michigan; m Division of Asthma Research, Cincinnati Children’s Hospital Medical Center, Cincinnati, Ohio; n Division of Clinical Immunology, Icahn School of Medicine at Mount Sinai, New York, New York; o Department of Pediatrics, Washington University School of Medicine, St. Louis, Missouri; p Division of Allergy and Clinical Immunology, Department of Internal Medicine, Henry Ford Health, Detroit, Michigan; q Department of Pediatrics, Vanderbilt University Medical Center, Nashville, Tennessee; r Division of Allergy, Department of Medicine, Pulmonary and Critical Care Medicine, Vanderbilt University School of Medicine, Nashville, Tennessee; s Division of Pulmonary and Critical Care, Department of Medicine, Brigham and Women’s Hospital and Harvard Medical School, Boston, Massachusetts

**Keywords:** Air pollution, Asthma, Childhood asthma, Water damage/home dampness, Pets, PM_2.5_

## Abstract

**Background:**

Children encounter multiple indoor and outdoor environmental exposures in early life. We assessed the independent effects of indoor home exposures and ambient particulate matter with an aerodynamic diameter ≤2.5 µm (PM_2.5_) on early childhood asthma diagnosis.

**Methods:**

We included 6,413 children born 1987–2016 from nine United States prospective birth cohorts from the Environmental Influences on Child Health Outcomes consortium, with complete covariate and outcome data. Exposures were (1) average ambient PM_2.5_ levels during the first 3 years of life, and (2) indoor home exposures, including water damage/home dampness during infancy/childhood, dogs/cats at home during infancy, dust mite allergen during infancy/childhood. Asthma was defined as caregiver-reported or doctor-diagnosed asthma anytime from birth to age 5. We applied Cox proportional hazards models, adjusting for individual-level and neighborhood-level confounders. Cohort-specific effects were implemented as fixed effects.

**Results:**

By age 5 years, 10.3%–50.3% of children had developed asthma across general-risk and high-risk cohorts. We found a significant detrimental association of PM_2.5_ and water damage/home dampness, and a protective association of dogs in the home with risk of childhood asthma, regardless of PM_2.5_ adjustment. The effect of having both water damage/home dampness and high PM_2.5_ on asthma diagnosis was greater than that of no water damage/home dampness and having low PM_2.5_ (hazard ratio: 1.95 [95% confidence interval = 1.19, 3.20]). There were no significant associations with household cats or dust mites.

**Conclusion:**

Multiple early exposures, such as PM_2.5_, home dampness, and absence of dogs in the home, should be considered together as risk factors for childhood asthma.

**What this study adds**

In this multicohort study, exposure to particulate matter with an aerodynamic diameter ≤2.5 µm, absence of dogs, and history of water damage or home dampness were independently associated with childhood asthma diagnosis. Multiple exposures, such as particulate matter with an aerodynamic diameter ≤2.5 µm, home dampness, and dogs in the home, should be considered in assessing the impact of risk and protective factors for childhood asthma.

## Introduction

The development of childhood asthma may be influenced by many environmental exposures from both indoor and outdoor sources. Children encounter multiple exposures that, together, may combine or interact to amplify or reduce the risk of asthma. Many prior individual cohort studies have had limited power to consider the effects of multiple indoor as well as outdoor environmental exposures. The Environmental Influences on Child Health Outcomes (ECHO) study is a large geographically, socially, and economically diverse consortium of longitudinal cohorts across the United States. A subset of participating cohorts collected data on early life indoor and outdoor environmental exposures that were considered potential risk factors for childhood asthma diagnosis.^1,2^

Within individual cohort studies participating in ECHO, we and others have previously demonstrated that indoor home exposures, such as dogs or cats in the home, dust mite allergen levels in the child’s bed in infancy or early life, and water damage/home dampness, are associated with early life respiratory symptoms/illnesses.^3–9^ We recently harmonized and pooled data from ECHO cohorts and confirmed that greater early life exposure to outdoor particulate matter with an aerodynamic diameter ≤2.5 µm (PM_2.5_) was linked to a higher risk of asthma diagnosis.^10–12^ In follow-up, pooling of data from multiple ECHO birth cohorts (eTable 1; https://links.lww.com/EE/A388) has provided the additional opportunity to test whether indoor home exposures and outdoor PM_2.5_ have independent, cumulative, or interacting effects on asthma risk to address the reality that children encounter multiple indoor and outdoor environmental factors in early childhood.

## Methods

### Study design and population

**Figure 1.**
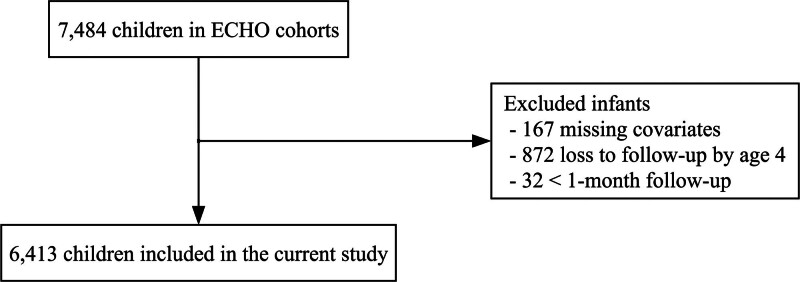
Flow diagram of participant selection. Among 7,484 infants from nine ECHO cohorts, we included 6,300 infants who had complete data for the covariates (infant sex, race and ethnicity, parental history of asthma, maternal education, and maternal smoking during pregnancy), neighborhood-level confounders (% population with low income and % Black population), and childhood asthma outcome. ECHO indicates Environmental Influences on Child Health Outcomes.

We hypothesized that indoor home exposures contribute to the development of childhood asthma independently of PM_2.5_ exposure. We included children and their families from nine longitudinal ECHO birth cohorts in the United States (eight cohorts in the Children’s Allergy & Asthma Data Repository [CADRE] and Project Viva).^1,2^ Four cohorts were asthma high risk cohorts based on parental history of asthma or allergy as an inclusion criterion (Cincinnati Childhood Allergy and Air Pollution Study, Childhood Origins of Asthma study, The Epidemiology of Home Allergens and Asthma Study [EHAAS], and Urban Environment and Childhood Asthma study [URECA]), and five cohorts were from the general population (Children’s Asthma Study [CAS], Columbia Center for Children’s Environmental Health Cohort, Infant Immune Study, Wayne County Health Environment Allergy and Asthma Longitudinal Study, and Project Viva). Participant births were from 1987 to 2016. The inclusion and exclusion criteria for the nine birth cohorts are summarized in eTable 1; https://links.lww.com/EE/A388. Of 7,484 children from the nine cohorts who were followed longitudinally beginning in early childhood for incident asthma, we excluded those (1) who were not followed beyond 1 month of age and (2) who did not have data on the covariates we adjusted for in the main analyses (child’s sex, race, ethnicity, parental history of asthma, maternal education, maternal smoking during pregnancy, % low-income neighborhood, and % Black, non-Hispanic neighborhood) (Figure 1). Our study was approved by the Institutional Review Board at each institution. Written informed consent was provided by a child’s parent or legal guardian. We report this study according to the Strengthening the Reporting of Observational Studies in Epidemiology Statement.^13^

### Exposures

We focused on early childhood exposures, as early life has been identified as a time window of lung and immune system development and heightened susceptibility to environmental exposures.^14^ Our primary indoor home exposures of interest included: the earliest postnatal questionnaire-based report of: (1) water damage/home dampness during infancy or childhood (harmonizing across cohorts reports of water damage in the home and reports of moisture or mildew in the house/on ceiling, walls or windows); (2) dog in the home during infancy (defined as 46 weeks post last menstrual period to infant age 11 months, 30 days); and (3) cat in the home during infancy. We also evaluated (4) the earliest postnatal child bed/crib or bedroom floor dust mite allergen levels of Der f 1 and/or Der p 1 μg/g in dust collected according to standard protocols from the child’s bedroom bed or floor during infancy or early childhood. Collection protocols differed by cohort, and bedroom dust mite data harmonized across cohorts represented either a mixed bedroom bed and floor sample (CAS, Project Viva, and URECA); the highest value from the bedroom floor or bed (EHAAS), or the bed sample alone (Columbia Center for Children’s Environmental Health Cohort), measured by ELISA. In Supplementary File 2; https://links.lww.com/EE/A389, we report the median age of questionnaire reports of water damage/home dampness and of dust sample collection by exposure and cohort. Water damage/home dampness, dog in the home, and cat in the home were categorized as yes/no variables. Dust mite was categorized into two groups (<10 µg/g or ≥10 µg/g of Der f 1 or Der p 1).^6^

Our primary outdoor exposure was estimated outdoor (ambient) PM_2.5_ linked to the longitudinal participant residential addresses from birth through the first 3 years of life. We focused on this exposure average, not only because it represented early childhood exposure but also because it was strongly associated with incident asthma in CADRE birth cohorts.^12^ The daily estimates of PM_2.5_ were derived from previously validated predictive models.^15–17^ In short, we used the model developed by Yanosky et al. between 1988 and 2006, and that by Schwartz et al. between 2000 and 2016, then calibrated these two models based on the overlapping relationships. To estimate annual averages of PM_2.5_ over the first 3 years of life, taking into account move histories, we created a longitudinal history of each participant’s addresses from birth to before 3 years of age, geocoded those addresses, and appended daily estimates of pollution to the longitudinal histories of geocodes. Yanoski et al. estimated monthly PM_2.5_ at the 6 km2 grid by using a spatiotemporal generalized additive mixed model incorporating records of PM_2.5_ on the monitoring sites, the Interagency Monitoring of Protected Visual Environments network, geographic coordinates at monitoring sites, a geographic information system, and meteorological data.^15^ Di et al. estimated daily PM_2.5_ at the 1 km2 grid by a neural network model incorporating records of PM_2.5_ on the monitoring sites, satellite-derived data, output data from a chemical transport model, geographic information system, and meteorological data.^16,17^ Children born in 1987 (226 out of 6,413 [3.5%]) were assigned PM_2.5_ exposure from age 2 to before 3, as Yanoski’s model does not include data for the year 1987.

### Outcomes

Our primary outcome of interest was the time to first report of childhood asthma diagnosis (in months) from birth to before 5 years of age. Secondarily, we considered first report of childhood asthma diagnosis (in months) from birth to before 12 years of age. Childhood asthma was captured by a questionnaire as a caregiver report of asthma diagnosed by a doctor or health care provider, and each cohort provided the timing of the first asthma diagnosis (eTable 2; https://links.lww.com/EE/A388). The follow-up period for each subject was defined as the months from the initiation of follow-up to the occurrence of either the first diagnosed asthma or the last recorded observation, whichever occurred first. The timing of collecting the questionnaire depended on the time of the follow-up visit in each cohort. We focused on the early childhood period because early childhood has been defined as a period of heightened vulnerability to environmental exposures with respiratory impairment or immune alterations that may persist into mid-childhood and adulthood.^18,19^ Children were included in the analysis if their asthma status was assessed at least once before reaching age 5 years and if the total duration of follow-up was more than 0 months. As sensitivity analyses, we assessed (1) the time to childhood asthma diagnosis (in months) before age 12 years, defined as a positive response to the asthma questionnaire among children who completed at least one questionnaire before age 12, and (2) the time to childhood asthma diagnosis which required any wheeze at age 3 years or above (evidence for asthma symptoms after infancy). In the second sensitivity analysis, children who had asthma and were not followed up until age 3 years were censored.

### Statistical analysis

In our descriptive analyses, we described participant characteristics and primary indoor home exposures (Table 1) for each cohort as proportions for categorical variables and as median with interquartile range for continuous variables. The distribution of PM_2.5_ across the cohorts was summarized as 10th and 90th percentiles, a value also used to scale estimates of pollution effects.

**Table 1.** Child and parent study characteristics, by cohort

	CCCEH (New York, NY) (N = 671)	IIS (Tucson, AZ) (N = 454)	COAST (Madison, WI) (N = 251)	CCAAPS (Cincinnati, OH) (N = 650)	EHAAS (Cambridge, MA) (N = 487)	WHEALS (Detroit, MI) (N = 1,105)	CAS (Detroit, MI) (N = 750)	Project Viva (Boston, MA) (N = 1,564)	URECA (Baltimore, MD) (N = 143)	URECA (Boston, MA) (N = 108)	URECA (New York, NY) (N = 87)	URECA (St. Louis, MO) (N = 143)	Total (N = 6,413)
High risk of asthma as an inclusion criterion^a^	No	No	Yes	Yes	Yes	No	No	No	Yes	Yes	Yes	Yes	–
Child’s sex (Female, %)	51.3	51.5	43.4	46.6	46.6	50.2	51.3	47.9	47.6	47.2	47.1	51.0	48.9
Child race and ethnicity (%)													
White	0.0	66.7	91.6	73.7	79.5	28.6	91.3	66.2	2.1	4.6	0.0	2.8	53.8
Hispanic	64.2	25.3	2.8	1.4	7.6	6.2	2.7	4.9	1.4	29.6	60.9	2.1	13.3
Black	35.8	1.8	4.0	19.2	6.8	60.8	1.5	14.7	90.9	54.6	32.2	92.3	26.2
Other	0.0	6.2	1.6	5.7	6.2	4.3	4.5	14.3	5.6	11.1	6.9	2.8	6.8
Parental history of asthma (%)	28.8	35.7	66.1	42.3	54.2	37.9	28.9	23.3	65.0	73.1	75.9	68.5	37.4
Maternal education (%)													
No HS diploma	35.9	6.2	1.6	6.6	1.2	5.6	3.2	2.2	55.9	48.1	57.5	53.1	10.9
HS diploma or some college	55.3	68.7	26.3	43.5	20.1	64.5	64.5	29.5	44.1	51.9	42.5	46.9	47.0
Bachelor’s degree/technical degree or graduate	8.8	25.1	72.1	49.8	78.6	29.9	32.3	68.2	0.0	0.0	0.0	0.0	42.1
Maternal smoking during pregnancy (%)	2.1	10.1	4.8	11.8	6.2	11.4	16.4	11.3	17.5	21.3	6.9	23.1	10.8
Maternal smoking during infancy (%)	14.8	11.2	6.8	11.7	4.9	14.6	20.0	4.5	42.7	34.3	27.6	48.3	13.1
Daycare attendance during infancy (%)	12.8	47.4	54.2	34.0	44.6	57.8	36.1	19.2	25.2	29.6	17.2	39.9	34.7
Cesarian section delivery (%)	23.0	22.7	13.9	0.0	23.0	37.1	18.9	23.3	31.5	37.0	27.6	24.5	22.8
Ever breastfed during infancy (%)	73.8	89.9	80.5	71.1	75.2	73.5	56.8	85.0	35.0	80.6	67.8	46.9	74.3
Number of children born to the mother (median [IQR])	1.00 (0.00, 2.00)	1.00 (1.00, 2.00)	–	2.00 (1.00, 3.00)	2.00 (1.00, 2.00)	2.00 (1.00, 3.00)	1.00 (1.00, 2.00)	2.00 (1.00, 2.00)	2.00 (1.00, 3.00)	2.00 (1.00, 3.00)	2.00 (1.00, 3.00)	2.00 (1.00, 3.00)	2.00 (1.00, 2.00)
% low income (median of *Z* score [IQR])	1.45 (1.06, 1.90)	−0.13 (−0.96, 0.69)	−0.77 (−1.05, −0.37)	−0.50 (−0.93, 0.10)	−0.33 (−0.85, 0.36)	0.33 (−0.47, 1.22)	−0.09 (−0.80, 0.48)	−0.57 (−0.91, −0.12)	1.15 (0.45, 1.88)	0.64 (0.05, 1.27)	1.54 (0.98, 2.07)	1.04 (0.75, 1.79)	−0.09 (−0.75, 0.97)
% Black, non-Hispanic (median of *Z* score [IQR])	0.87 (0.07, 2.39)	−0.44 (−0.51, −0.38)	−0.51 (−0.56, −0.38)	−0.47 (−0.54, 0.35)	−0.48 (−0.52, −0.33)	1.91 (−0.36, 3.49)	−0.53 (−0.54, −0.49)	−0.48 (−0.54, 0.04)	3.39 (2.76, 3.59)	1.77 (0.21, 2.58)	0.99 (0.67, 1.73)	3.38 (1.82, 3.66)	−0.38 (−0.53, 1.11)
Population density per 1 km2 at birth address (median of *Z* score [IQR])	9.66 (6.19, 12.84)	−0.11 (−0.34, 0.00)	−0.32 (−0.41, −0.13)	−0.19 (−0.31, −0.02)	0.08 (−0.15, 0.75)	0.07 (−0.12, 0.25)	−0.09 (−0.27, 0.07)	−0.01 (−0.30, 0.80)	0.97 (0.41, 1.48)	1.28 (0.72, 1.95)	7.23 (4.33, 9.02)	0.06 (−0.16, 0.25)	0.03 (−0.22, 0.58)
Water damage/home dampness (%)	19.8	0.0	0.0	43.1	31.8	9.3	0.0	35.3	13.3	34.3	34.5	27.3	21.0
Dog at home during infancy (%)	5.1	57.5	36.3	35.1	16.2	25.9	34.1	17.7	21.7	10.2	21.8	21.7	25.0
Cat at home during infancy (%)	9.5	33.3	30.3	22.8	21.8	15.5	20.3	24.5	42.0	31.5	27.6	16.1	21.7
Dust mite (Der f 1 or Der p 1) allergen in the child bed or bedroom floor (%)													
≤10 µg/g	71.7	0.0	0.0	0.0	67.6	0.0	10.5	11.3	90.2	98.1	90.8	97.2	23.7
>10 µg/g	2.1	0.0	0.0	0.0	30.6	0.0	1.1	7.0	0.0	0.0	0.0	0.7	4.4
Asthma cumulative incidence by age 5 (%)	31.1	13.7	21.5	12.5	27.3	16.6	10.3	15.3	34.3	46.3	43.7	50.3	19.4
Asthma cumulative incidence by age 11 (%)	39.8	16.1	43.0	19.1	33.5	17.8	16.3	27.5	38.5	49.1	49.4	59.4	26.8

a High-risk cohorts were determined based on the parental history of asthma or allergies as an inclusion criteria.

CAS indicates Children’s Asthma Study; CCAAPS, Cincinnati Childhood Allergy and Air Pollution Study; CCCEH, Columbia Center for Children’s Environmental Health Cohort; COAST, Childhood Origins of Asthma study; EHAAS, The Epidemiology of Home Allergens and Asthma Study; HS, high school; IIS, Infant Immune Study; IQR, interquartile range; URECA, Urban Environment and Childhood Asthma study; WHEALS, Wayne County Health Environment Allergy and Asthma Longitudinal Study.

We used the multivariable Cox proportional hazards model to evaluate, over all cohorts in our harmonized data sets, the effects of PM_2.5_ and indoor home exposures asthma first diagnosed in early childhood (< age 5) and, secondarily, on asthma diagnosed by mid-childhood (<age 12). (1) First, adjusting for the covariates described below, we evaluated the single-exposure effects of PM_2.5_ and indoor home exposures by including each primary exposure in the models one at a time. We calculated the effect size of PM_2.5_ by multiplying by 6.74 to reflect the change from the 10th to 90th percentile of the entire cohort. Adjusted hazard ratios were calculated by exponentiating the regression coefficients. (2) Second, in dual primary exposure models, we evaluated the effect of each indoor home exposure, independent of the effect of PM_2.5_, by additionally adjusting for PM_2.5_, with no interaction term. In the model including PM_2.5_ and home dampness/water damage, we calculated an overall estimate of exposure burden by summing the coefficients of PM_2.5_ and indoor home exposures. Specifically, we calculated the combined adjusted hazard ratio comparing the presence of home dampness/water damage at the 90th percentile of PM_2.5_ versus the absence of home dampness/water damage at the 10th percentile of PM_2.5_. This represents the linear combination of regression coefficients of the history of water damage/home dampness and the 90–10th percentile change in PM_2.5_. (3) Third, we evaluated the interaction of PM_2.5_ with each indoor home exposure, one at a time, by including a multiplicative interaction term for each interaction we considered. In exploratory analyses, we also assessed the effect measure modification of a parental history of asthma (yes vs. no) and cohort risk status (high-risk vs. general-risk) based on the parental history of asthma or allergy on the association between each exposure and childhood asthma.

All the models were adjusted for clinically important potential confounders and predictors of asthma based on previous literature and our previously published papers on predictors of incident asthma in CADRE and Project Viva.^9,12,20^ Individual-level confounders included child race and ethnicity (White, Hispanic, African American, and other), parental history of asthma (either maternal or paternal history of asthma vs. not), maternal education (no high school diploma, high school diploma or some college, bachelor’s degree/technical degree or graduate), maternal smoking during pregnancy (yes vs. no). The child’s and parents’ confounders and predictors of asthma were collected by using questionnaire data collected at baseline. Neighborhood-level confounders (i.e., % population with low income and % Black population) were obtained at the census-tract level from the U.S. Census for the years 1980, 1990, 2000, and 2010 based on each participant’s birth record address by the Harvard Center for Geographic Analysis. These census variables from the year closest to the child’s birth year were linked. Neighborhood-level variables were converted to z-scores by subtracting the nationwide mean of all tract values from the value for that tract and dividing this result by the standard deviation of all tract values. The baseline hazards were stratified by child’s sex (male vs. female). In addition, cohort-specific effects were implemented as a fixed-effect and included in the statistical models as categorical variables.

We conducted complete case analyses because most missing data were related to exposure and outcome measures in the original cohort, while missing covariate data were relatively rare (305/7,484 [4%], Figure 1). Therefore, we did not perform multiple imputation and excluded children with missing covariate or outcome data as part of the exclusion criteria. R software version 4.5.1 (R Foundation for Statistical Computing, Vienna, Austria) was used.

## Results

### Descriptive analysis

**Figure 2.**
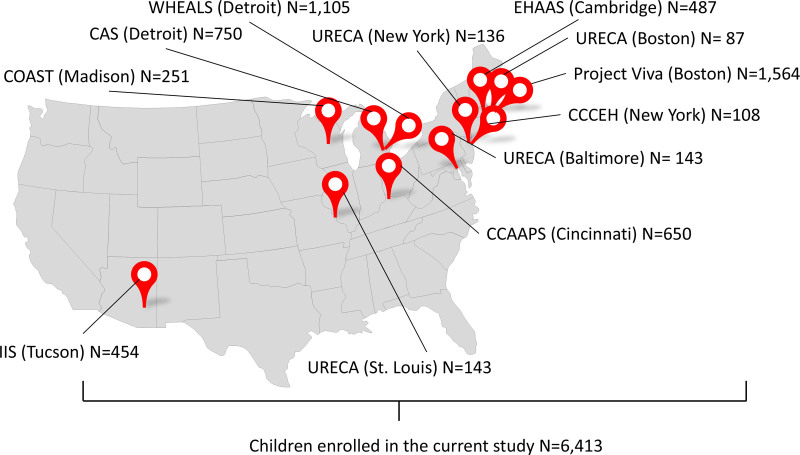
Geographical distribution of the included cohorts. CAS indicates Children’s Asthma Study; CCAAPS, Cincinnati Childhood Allergy and Air Pollution Study; CCCEH, Columbia Center for Children’s Environmental Health Cohort; COAST, Childhood Origins of Asthma study; EHAAS, The Epidemiology of Home Allergens and Asthma Study; IIS, Infant Immune Study; URECA, Urban Environment and Childhood Asthma study; WHEALS, Wayne County Health Environment Allergy and Asthma Longitudinal.

We included a total of 6,413 children enrolled in the ECHO consortium from nine birth cohorts with complete data for covariates/potential confounders previously shown in the CADRE consortium to be predictive of asthma incidence (Figure 2).^9,12^ The number of children included varied across statistical analyses depending on the availability of primary exposure data. The child and parent study characteristics were heterogeneous (Table 1 and eTable 4; https://links.lww.com/EE/A388). Across cohorts, the 10th percentile of PM_2.5_ exposure in the first 3 years was 9.21 µg/m^3,^ and the 90th was 15.9 µg/m^3^. By age 5 years, 10.3 to 50.3% (10.3%–31.1% in the general-risk cohorts, and 12.7%–50.3% in the high-risk cohorts) of children developed asthma (Table 1).

### Statistical analysis results

Table 2 summarizes the estimated exposure effects of PM_2.5_ and indoor home exposures from single-exposure models, and Table 3 summarizes the estimated independent effects from two exposure models (each pair of PM_2.5_ and an indoor home exposure), both tables adjusted for potential confounders. While outdoor air pollution estimates were available for all cohorts, the number of observations for analyses varied by which cohorts ascertained specific indoor home exposures (eTables 2 and 3; https://links.lww.com/EE/A388). In single-exposure models, PM_2.5_ and water damage/home dampness had detrimental associations with childhood asthma diagnosis (PM_2.5_: hazard ratio [HR] for 10–90th percentile change = 1.49, 95% confidence interval [CI] = 1.01, 2.17; history of water damage/home dampness: HR = 1.17, 95% CI = 1.02, 1.35). The presence of dogs at home during infancy had a protective association with childhood asthma (HR = 0.81, 95% CI = 0.70, 0.94) (Table 2). There were no significant associations of cats in the home (HR = 0.93, 95% CI = 0.81, 1.08) or dust mite exposure (HR = 1.20, 95% CI = 0.89, 1.61) in the child’s bed or bedroom with risk of asthma. In models adjusted for PM_2.5_, the associations of indoor home exposures with asthma risk were in the same direction (Table 3).

**Table 2.** Summary results of associations of single-exposure models on childhood asthma diagnosis before age 5

Exposures	Number of subjects	Number of asthma outcomes (%)	Hazard ratio	95% confidence interval	p-value
Single-exposure effect (without adjustment for PM_2.5_)					
PM_2.5_	5,480	1,085 (19.8)	1.48	1.01–2.17	0.04
Water damage/home dampness	4,760	1,012 (21.3)	1.17	1.02–1.35	0.03
Dogs at home	5,707	1,105 (19.4)	0.81	0.70–0.94	0.01
Cats at home	5,719	1,115 (19.5)	0.93	0.81–1.08	0.34
Dust mite (Der f 1 or Der p 1) allergen in the child bed or bedroom floor	1,801	536 (29.8)	1.20	0.89–1.61	0.24

Cox proportional hazards models were used to evaluate the single-exposure effects of PM_2.5_ and indoor home exposures by including each of them in the model one at a time. Child’s sex, race, ethnicity, parental history of asthma, maternal education, maternal smoking during pregnancy, % low-income neighborhood (*Z* score), and % Black, non-Hispanic neighborhood (*Z* score) were adjusted as covariates. Cohort-specific effects were implemented as fixed effects. PM_2.5_ was scaled to reflect the change from the 10th to 90th percentile of the entire cohort (6.74 µg/m^3^).

N indicates number; PM_2.5_, particulate matter with an aerodynamic diameter ≤2.5 µm.

**Table 3.** Summary results of dual-exposure models on childhood asthma diagnosis before age 5

Exposures	Number of subjects	Number of asthma outcomes (%)	Hazard ratio of an indoor exposure	95% confidence interval	*P*-value	Hazard ratio of PM_2.5_	95% confidence interval	*P*-value
Independent exposure effect (with adjustment for PM_2.5_)								
Water damage/home dampness	4,067	880 (21.6)	1.13	0.98–1.32	0.10	1.72	1.07–2.75	0.02
Dogs at home	5,068	984 (19.4)	0.80	0.68–0.94	0.01	1.45	0.97–2.17	0.07
Cats at home	5,086	994 (19.5)	0.93	0.79–1.08	0.31	1.45	0.97–2.16	0.07
Dust mite (Der f 1 or Der p 1) allergen in the child bed or bedroom floor	1,534	456 (29.7)	1.17	0.87–1.59	0.30	1.53	0.76–3.11	0.24

Cox proportional hazards models were used to evaluate the independent exposure effects of PM_2.5_ and each indoor home exposure by including each pair in the model one at a time. Childs’s sex, race, ethnicity, parental history of asthma, maternal education, maternal smoking during pregnancy, % low-income neighborhood (*Z* score), and % Black, non-Hispanic neighborhood (*Z* score) were included as covariates. Cohort-specific effects were implemented as fixed effects. PM_2.5_ was scaled to reflect the change from the 10th to 90th percentile of the entire cohort (6.74 µg/m^3^).

N indicates number; PM_2.5_, particulate matter with an aerodynamic diameter ≤2.5 µm.

Using the Cox proportional hazards model without an interaction term, we analyzed the combined association of water damage/home dampness and the 10–90th percentile change in PM_2.5_ with childhood asthma. The hazard ratio for both the presence of both water damage/home dampness and high PM_2.5_ (90th percentile) was 1.95 (95% CI = 1.19, 3.20), relative to having no water damage and low PM_2.5_ (10th percentile). We did not find any significant pairwise interactions between PM_2.5_ and indoor home exposures (PM_2.5_ and water damage/home dampness: *P* for interaction = 0.63; PM_2.5_ and dogs: *P* for interaction = 0.32; PM_2.5_ and cats: *P* for interaction = 0.51; PM_2.5_ and dust mites: *P* for interaction = 0.68). Parental history of asthma or cohort status did not modify the association of PM_2.5_, water damage/home dampness, or dog exposure in early life with asthma diagnosis (eTables 5 and 6; https://links.lww.com/EE/A388). In sensitivity analyses, we assessed (1) the time to childhood asthma diagnosis (in months) before age 12 years (2) the time to childhood asthma diagnosis and report of any wheeze at age 3 or above. The results were similar to those from the primary analyses (eTables 7 and 8; https://links.lww.com/EE/A388).

## Discussion

While individual cohort findings have been seminal in demonstrating important environmental contributions to asthma, harmonization of extant data from the diverse ECHO cohorts presented the opportunity to further evaluate the independent and combined effects of multiple exposures and their sources on childhood asthma risk.^3,4,6,9,10,18,21–23^ In this longitudinal study with harmonized data from nine U.S. birth cohorts, ambient PM_2.5_ and indoor home water damage/home dampness together in early childhood were associated with an increased risk of the diagnosis of childhood asthma before 5 years of life, whereas the presence of dogs in the home was associated with a reduced risk of early childhood asthma after adjusting for potential individual- and neighborhood-level confounders and risk factors for asthma. The directions of both individual indoor home exposures with asthma remained the same after adjusting for PM_2.5_, which itself remained a risk factor. The estimate of effects of both exposure to both ambient high-level PM_2.5_ in the first 3 years of life, plus indoor water damage/home dampness on asthma diagnosis in early childhood was greater than the individual estimates for either adverse exposure.

Previously the Cincinnati Childhood Allergy and Air Pollution Study cohort—based in Cincinnati and a birth cohort that is part of ECHO and CADRE—found that the presence of three molds in homes with water damage during early childhood was associated with a higher prevalence of asthma at age 7.^24^ EHAAS, a Boston cohort that is also now part of ECHO and CADRE, found early fungal exposures influenced allergic rhinitis by age 5, but not asthma risk.^7^ A strength of this analysis is that evidence for home water damage/home dampness increasing risk of asthma diagnosis was strengthened by harmonizing multiple birth cohorts. Single-cohort literature has consistently supported that home water damage or water damage-associated molds are related to child respiratory illness/infections and asthma exacerbations.^25^ The harmonized ECHO birth cohorts provided increased power, greater generalizability by including children across four decades and from different geographic areas in the United States, and by including both high-asthma-risk and general-risk populations.^1,2^ Moreover, our findings underscore the importance of considering both air pollution outside the home and indoor home environmental exposures together when evaluating environmental influences on asthma diagnosis and designing prevention strategies to reduce asthma risk.

Birth cohorts included in ECHO and CADRE, including CAS and Wayne County Health Environment Allergy and Asthma Longitudinal Study (Detroit), Childhood Origins of Asthma study (Madison), and URECA (four urban locations), have previously reported that early life dog and/or cat exposures were associated with lower allergic sensitization and allergic diseases.^9,23,26,27^ Another ECHO and CADRE birth cohort, EHAAS, has previously found dog and endotoxin—but not cat—protective of allergy and asthma, with cat or cat allergen effects potentially modified by maternal asthma history.^3,4^ In our harmonized multicohort analysis, dog was consistently and inversely associated with asthma, whereas cat was not.

### Study strengths and limitations

Our study has many strengths, including the prospective study design and large population. Questionnaires on cat and dog ownership were gathered during infancy, and the majority of questionnaires on water damage/home dampness were also collected within the first year of life (eTable 2; https://links.lww.com/EE/A388). The timing of ascertainment of exposures and outcome minimized the risk of reverse causation, a common limitation in cross-sectional studies, but did not eliminate the possibility of uncertainty in the temporal relationship between exposures and outcomes. Our study approach had additional potential limitations. While most cohorts ascertained diagnosis of asthma by doctors or health care providers with similar standard questions, the threshold for diagnosing repeated respiratory symptoms or impairment as asthma varies by community and over time.^28^ A significant proportion of asthma diagnoses occurred before the age of 4–5 years.^21^ Diagnosing asthma at such an early age is inherently challenging, potentially leading to outcome misclassification. Because we used a survival analysis framework with time to asthma diagnosis as the outcome, we were unable to directly address this issue. Nevertheless, asthma diagnosed in early childhood has been predictive of later childhood respiratory symptoms and lung function impairment, motivating efforts for early intervention.^29^

Harmonization of exposure and outcome data has strengths compared to meta-analysis, but also limitations. Harmonization of exposures ascertained across cohorts with differing questions (e.g., water damage/home dampness, Supplementary File 2; https://links.lww.com/EE/A389) or sampling schemas (e.g., dust mite, Supplementary File 2; https://links.lww.com/EE/A389) meant that we were unable to use the same measures ascertained at the same intervals across all cohorts. Thus, we applied post hoc definitions to ensure comparability, while allowing some flexibility. This approach may also have introduced some exposure misclassification. In addition, with the exception of PM_2.5_, with our emphasis on characterizing early childhood exposure relationship to asthma, our exposures were measured at a single time point, without considering temporal changes in exposures or cumulative exposure. While also emphasizing the effects of early life environment on asthma risk, some previous studies have suggested that the timing of pet exposure, and variations in how dogs and cats are cared for, may differentially influence asthma development.^30,31^ While our harmonized analyses demonstrate a non-significant association between the presence of dust mite exposure and early asthma, the power to test this association was limited because few cohorts had child bedroom dust mite measures. Despite combining data across multiple cohorts, we still had relatively low power for going beyond dual-exposure modeling and assessing interactions of outdoor (PM_2.5_) exposure with our primary indoor exposures. Our statistical approach using fixed rather than random effects modeling resulted in an overall estimate of the association of each exposure with diagnosed asthma, but the opportunity to evaluate between-cohort differences in exposure effects was limited by some modest cohort-specific sample sizes and, therefore, low power.^32^ We chose fixed cohort effects models because they have the advantage over random cohort effects models of removing the possibility of between-cohort confounding of our effects of interest. Moreover, because the populations within each cohort are very different, with a large number of factors differing among these populations, we felt the potential for between-cohort confounding was high, and so we took the more conservative modeling approach to ensure we adjusted for these factors.

## Conclusion

Among demographically and geographically diverse cohorts with births over several decades, we demonstrated the independent and combined effects of early life PM_2.5_ and water damage/home dampness with increased risk of childhood asthma. We also demonstrated that dogs in the home are protective for asthma. Our study results support the assessment of multiple exposures, such as PM_2.5_, home dampness, and the absence of dogs in the home, together as risk factors for asthma.

## Conflicts of interest statement

A.Z. reported grants from the National Institutes of Health (NIH) during the conduct of the study and grants from the National Institute on Aging outside the submitted work. B.A.C. reported grants from NIH during the conduct of the study, and from Apple, Inc., outside the submitted work. P.H.R., C.C.J., R.L.M., D.R.G., and E.M.Z. reported grants from NIH during the conduct of the study. J.E.G. reported grants from NIH during the conduct of the study, personal fees from Arrowhead Pharmaceuticals, AstraZeneca, and Meissa Vaccines, Inc., and stock options for Meissa Vaccines, Inc. outside the submitted work. S.K.R. reported receiving consulting fees from Sanofi. P.I.B. reported grants from NIH during the conduct of the study and grants from NIH and the Environmental Protection Agency outside the submitted work. D.J.J. reported grants from NIH during the conduct of the study and grants and/or personal fees from Areteia, Astra Zeneca, Avillion, GlaxoSmithKline, Sanofi, Regeneron, Genentech, Pfizer, Upstream Bio, outside the submitted work. F.D.M. reported consultancy fees from OM PHARMA. T.V.H. reported grants from NIH and the World Health Organization during the conduct of the study and personal fees from the American Thoracic Society, speaker honorarium from the American Academy of Allergy, Asthma and Immunology, the Parker B. Francis Foundation Scientific Advisory Board, and Pfizer outside the submitted work. Other authors had nothing to disclose.

## Acknowledgments

The authors wish to thank our ECHO Colleagues; the medical, nursing, and program staff; and the children and families participating in the ECHO cohort.

## Supplementary Material


