# Supplementary material for: Individual and combined effects of indoor home exposures and ambient PM2.5 during early life on childhood asthma in us birth cohort studies
Source: Environ Epidemiol. 2025 Dec 23;10(1):e443. doi: 10.1097/EE9.0000000000000443 (PMC12737862; doi:10.1097/EE9.0000000000000443)
Supplement: Supplementary file 2 [file ee9-10-e443-s002.pdf]

Online-only material

Methods

eTable 1. Eligibility criteria for each cohort

eTable 2. Timing of the questionnaires collecting home dampness/water damage, dust mite, and asthma variables in each cohort

eTable 3. Cohorts included in each statistical analysis

eTable 4. Missing data in child and parent study characteristics

eTable 5. Summary results of the single-exposure models on childhood asthma diagnosis before age 5 including an interaction with a parental history of asthma

eTable 6. Summary results of the single-exposure models on childhood asthma diagnosis before age 5 including an interaction with high-risk cohort status

eTable 7. Summary results of the sensitivity analysis for the outcome (single-exposure models)

eTable 8. Summary results of the sensitivity analysis for the outcome (dual-exposure models)

eTable 1. Eligibility criteria for each cohort

Cohort name	Inclusion criteria of the original study	Exclusion criteria of the original study	Recruitment strategy	Recruitment period
CCCEH (New York, NY)	1. Dominican or African American<br>2. Lived in Manhattan or the South Bronx for at least 1 year<br>3. Between the ages of 18 and 35<br>4. Registered at a prenatal clinic by 20 weeks of pregnancy	1. Smoking during pregnancy<br>2. Hypertension<br>3. HIV<br>4. Illicit drug use<br>5. Diabetes	Volunteers sought in OB/GYN clinics	1998–2006
IIS (Tucson, AZ)	1. Healthy child born to a woman who planned to seek care for her child through one of 14 collaborating Tucson pediatricians<br>2. Spoke English<br>3. Had a telephone	1. Diagnosis of an immune deficiency condition in the mother<br>2. Plans to leave Tucson.	Pregnant women were recruited during prenatal visits to 10 obstetric and eight midwife practitioners at five sites in Tucson	1997–2003
COAST (Madison, WI)	1. At least one parent with (physician-diagnosed) asthma or allergy (one or more positive skin tests)<br>2. Cord blood sample within 2 minutes of birth	1. Preterm birth<br>2. Low birth weight<br>3. Respiratory distress at birth	Families (pregnant mothers) recruited through local hospitals/clinics	1998–2000
CCAAPS (Cincinnati, OH)	1. At least one Atopic parent<br>2. Within 400m of roadways with > 1000 trucks per day<br>3. Further than 1000m of roadways > 1000 trucks per day	1. Neither parent tests positive to SPT of 15 allergens<br>2. In grey zone between 400m and 1000m of major roadway	Potential subject list generated from birth records of children born Oct 2001 to July2003 with birth record addresses within exposed or unexposed area. Letters mailed to families with follow-up phone calls home visits as needed.	2001–2003

	4. Born Oct 2001 – July 2003			
EHAAS (Cambridge, MA)	1. Parental report of doctor's diagnosis of asthma hay fever or allergy to inhalational allergens<br>2. Maternal age >18 years<br>3. No intention to move in the next 12 months (so as to accurately characterize exposure),<br>4. Residence considered safe by the mother to visit during daytime hours,<br>5. Maternal ability to speak English or Spanish, and<br>6. Living within Route 128	1. Prematurity <36 weeks,<br>2. Major congenital abnormalities<br>3. Hospitalization in the neonatal intensive care unit	Screened in hospital after birth of the index child	1994–1996
WHEALS (Detroit, MI)	1. Attending one of five health system clinics in Western half of Wayne County Michigan<br>2. Lived contiguously defined zip codes<br>3. Intending to reside in area for at least 2 years<br>4. Speak English well enough to understand informed consent<br>5. Ages 21-29	Non-English speaking	Tried to recruit all pregnant women attending one of five clinics in Wayne County residing in a circumscribed geographical area in Wayne County	2003–2007
CAS (Detroit, MI)	1. Residence in northern, suburban Detroit defined by ZIP codes	1. Major neonatal health problems<br>2. Plans to move from area within 12 months of birth	Pregnant women asked to participate during prenatal visits at HMO clinics.	1987–1989

	2. Parents insured by HAP health Maintenance organization<br>3. Gestation ≥36 weeks<br>4. Cord serum collected for IgE measurement			
Project Viva (Boston, MA)	1. Less than 22 weeks pregnant at the time of enrollment<br>2. Receive prenatal care at one of the selected practices<br>3. Plan on delivering at one of two study hospitals<br>4. Be able to answer questionnaires in English.	1. Multiple gestation<br>2. Plans to move away before delivery<br>3. Plans to terminate the pregnancy	Recruited women seen for prenatal care at eight urban and suburban practices of a multi-specialty group practice in eastern Massachusetts	1999–2002
URECA (Boston, MA; URECA, New York, NY; Baltimore, MD; St. Louis, MO)	1. Planning to deliver at the study hospital<br>2. A parental history of asthma, allergic rhinitis (hay fever), or eczema (atopic dermatitis)<br>3. Reside in census tracts with at least 20% of the residents with income below the poverty level<br>4. Gestational age at delivery of ≥ 34 weeks<br>5. Suitable cord blood specimen must be obtained and processed to establish baseline cytokine secretion	1. Respiratory distress requiring intubation and ventilation for four or more hours<br>2. Respiratory distress requiring either supplemental oxygen or CPAP for four or more days<br>3. Pneumonia requiring antibiotic treatment for one week or more<br>4. Significant congenital anomalies<br>5. Maternal HIV infection at time of delivery	Advertisement in general population, volunteers sought in OB/GYN clinics	2004–2006

	6. Plans for the family to move out of the geographic area during the period of the study<br>7. Does not consent to all aspects of the study<br>8. Does not have access to a phone<br>9. Does not speak English (or Spanish at sites with Spanish - speaking staff)<br>10. Administration of palivizumab (Synagis) for RSV prophylaxis	

Abbreviations: CAS=Children's Asthma Study, CCAAPS=Cincinnati Childhood Allergy and Air Pollution Study, CCCEH=Columbia Center for Children's Environmental Health Cohort, EHAAS=The Epidemiology of Home Allergens and Asthma Study, URECA=Urban Environment and Childhood Asthma study, WHEALS = Wayne County Health Environment Allergy and Asthma Longitudinal Study

eTable 2. Timing of the collection of home dampness/water damage, dust mite, and asthma variables in each cohort

Cohort name	Median age(months) of measurement of home dampness/water damage	Location of sampling of dust mites	Median age (months) of measurement of dust mite allergen (Der f 1 or Der p 1)	Median age (months) of first diagnosed asthma
CCCEH (New York)	12	Bed	13	34
CCAAPS (Cincinnati)	7	Not collected	Not collected	37
EHAAS (Metropolitan Boston)	6	Bed	2	24
CAS (Detroit)	Not collected	Bedroom	25	72
WHEALS (Detroit)	0	Not collected	Not collected	12
Project Viva (Metropolitan Boston)	13	Bedroom	49	61
URECA (Boston, New York, Baltimore, St. Louis)	24	Bedroom	3	24

This table summarizes the timing of the questionnaires collecting home dampness/water damage, dust mite, and asthma variables in each cohort. As for home dampness/water damage, if the questionnaires were collected multiple times, we used the months of visits collecting “Yes” (positive) for the first time. If the questionnaires were collected multiple times and they were all “No” (negative), we used the months of the first visits. COAST and IIS did not collect home dampness or dust mite variables. Childhood asthma was defined based on parental report of a doctor’s diagnosis. Each cohort provided the timing of asthma diagnosis.

Abbreviations: CAS=Children’s Asthma Study, CCAAPS=Cincinnati Childhood Allergy and Air Pollution Study, CCCEH=Columbia Center for Children’s Environmental Health Cohort, EHAAS=The Epidemiology of Home Allergens and Asthma Study, URECA=Urban Environment and Childhood Asthma study, WHEALS = Wayne County Health Environment Allergy and Asthma Longitudinal Study

eTable 3. Cohorts included in each statistical analysis

Exposures	Included cohorts
Single-exposure effect (without adjustment for $PM_{2.5}$)	
$PM_{2.5}$	CCCEH, IIS, COAST, CCAAPS, EHAAS, WHEALS, CAS, Project Viva, URECA
Water damage/home dampness	CCCEH, CCAAPS, EHAAS, WHEALS, Project Viva, URECA
Dogs at home	CCCEH, IIS, COAST, CCAAPS, EHAAS, WHEALS, CAS, Project Viva, URECA
Cats at home	CCCEH, IIS, COAST, CCAAPS, EHAAS, WHEALS, CAS, Project Viva, URECA
Dust mite (Der f 1 or Der p 1) allergen in the child bed or bedroom floor	CCCEH, EHAAS, CAS, Project Viva, URECA
Dual-exposure models (with adjustment for $PM_{2.5}$) and interaction models	
Water damage/home dampness	CCCEH, CCAAPS, EHAAS, WHEALS, Project Viva, URECA
Dogs at home	CCCEH, IIS, COAST, CCAAPS, EHAAS, WHEALS, CAS, Project Viva, URECA
Cats at home	CCCEH, IIS, COAST, CCAAPS, EHAAS, WHEALS, CAS, Project Viva, URECA
Dust mite (Der f 1 or Der p 1) allergen in the child bed or bedroom floor	CCCEH, EHAAS, CAS, Project Viva, URECA

Abbreviations: CAS=Children's Asthma Study, CCAAPS=Cincinnati Childhood Allergy and Air Pollution Study, CCCEH=Columbia Center for Children's Environmental Health Cohort, COAST=Childhood Origins of Asthma study, EHAAS=The Epidemiology of Home Allergens and Asthma Study, IIS=Infant Immune Study, URECA=Urban Environment and Childhood Asthma study, WHEALS = Wayne County Health Environment Allergy and Asthma Longitudinal Study

eTable 4. Missing data in child and parent study characteristics

	CCCEH (New York, NY) (N=671)	IIS (Tucson, AZ) (N=454)	COAST (Madison, WI) (N=251)	CCAAPS (Cincinnati, OH) (N=650)	EHAAS (Cambridge, MA) (N=487)	WHEALS (Detroit, MI) (N=1,105)	CAS (Detroit, MI) (N=750)	Project Viva (Boston, MA) (N=1,564)	URECA (Baltimore, MD) (N=143)	URECA (Boston, MA) (N=108)	URECA (New York, NY) (N=87)	URECA (St. Louis, MO) (N=143)	Total (N=6,413)
Maternal smoking during infancy (%)	16.4	0.0	0.0	9.8	0.4	0.6	11.5	18.6	5.6	6.5	13.8	7.7	9.3
Daycare attendance during infancy (%)	45.0	2.2	0.0	1.1	0.0	27.1	12.4	17.9	2.8	3.7	10.3	4.9	15.8
Cesarian section delivery (%)	2.1	0.2	0.4	100.0	0.0	0.5	5.3	0.3	0.0	0.0	0.0	0.0	11.1
Ever breastfed during infancy (%)	1.6	3.1	0.0	0.2	0.0	5.7	11.9	4.2	2.1	1.9	5.7	2.8	4.0
Number of children born to the mother (%)	0	0.4	100	0.3	0	0	1.6	0.0	0.0	0.0	0.0	0.0	4.2

	CCCEH (New York, NY) (N=671)	IIS (Tucson, AZ) (N=454)	COAST (Madison, WI) (N=251)	CCAAPS (Cincinnati, OH) (N=650)	EHAAS (Cambridge, MA) (N=487)	WHEALS (Detroit, MI) (N=1,105)	CAS (Detroit, MI) (N=750)	Project Viva (Boston, MA) (N=1,564)	URECA (Baltimore, MD) (N=143)	URECA (Boston, MA) (N=108)	URECA (New York, NY) (N=87)	URECA (St. Louis, MO) (N=143)	Total (N=6,413)
Water damage/home dampness (%)	3.0	100.0	100.0	8.5	1.2	3.2	100.0	4.5	0.7	0.9	8.0	2.1	25.8
Dog at home during infancy (%)	36.5	0.0	0.0	0.0	0.6	14.0	11.5	12.5	2.1	2.8	10.3	4.2	11.0
Cat at home during infancy (%)	32.0	0.0	0.0	0.0	0.6	15.5	11.5	12.7	2.1	2.8	10.3	4.2	10.8
Dust mite (Der f 1 or Der p 1) allergen in the child bed or bedroom floor (%)	26.2	100.0	100.0	100.0	1.8	100.0	88.4	81.6	9.8	1.9	9.2	2.1	71.9

Abbreviations: CAS=Children's Asthma Study, CCAAPS=Cincinnati Childhood Allergy and Air Pollution Study, CCCEH=Columbia Center for Children's Environmental Health Cohort, COAST=Childhood Origins of Asthma study, EHAAS=The Epidemiology of Home Allergens and Asthma Study, IIS=Infant Immune Study, IQR = Interquartile range, HS = High school, URECA=Urban Environment and Childhood Asthma study, WHEALS = Wayne County Health Environment Allergy and Asthma Longitudinal Study

eTable 5. Summary results of the single-exposure models on childhood asthma diagnosis before age 5 including an interaction with a parental history of asthma

Exposures	Number of subjects	Number of asthma outcomes (%)	Number of children with a parental history of asthma	Presence or absence of a parental history of asthma	Subgroup-specific hazard ratio	95% confidence interval
Single-exposure effect (with an interaction term with a parental history of asthma)						
$PM_{2.5}$	5,480	1,085 (19.8)	2,088	Presence	1.54	1.01–2.34
				Absence	1.44	0.95–2.18
Water damage/home dampness	4,760	1,012 (21.3)	1,762	Presence	1.22	1.02–1.47
				Absence	1.11	0.90–1.37
Dogs at home	5,707	1,105 (19.4)	2,157	Presence	0.76	0.62–0.94
				Absence	0.87	0.70–1.08
Cats at home	5,719	1,115 (19.5)	2,153	Presence	0.96	0.80–1.17
				Absence	0.89	0.71–1.11
Dust mite (Der f 1 or Der p 1) allergen in the child bed or bedroom floor	1,801	536 (29.8)	792	Presence	1.15	0.77–1.72
				Absence	1.24	0.84–1.83

Cox proportional hazards models were used to evaluate the single-exposure effects of $PM_{2.5}$ and indoor home exposures by including each of them in the model one at a time. Child's sex, race, ethnicity, parental history of asthma, maternal education, maternal smoking during pregnancy, % low-income neighborhood (Z score), and % Black, non-Hispanic neighborhood (Z score) were adjusted as covariates. Cohort-specific effects were implemented as fixed-effects. An interaction term between each exposure and a parental history of asthma was added to the models. $PM_{2.5}$ was scaled to reflect the change from the 10th to 90th percentile of the entire cohort (6.74 $\mu g/m^3$). We calculated the linear combination of regression coefficients of each exposure among those with and without a parental history of asthma.

Abbreviation: N=number, $PM_{2.5}$=particulate matter with an aerodynamic diameter ≤2.5 µm

eTable 6. Summary results of the single-exposure models on childhood asthma diagnosis before age 5 including an interaction with high-risk cohort status

Exposures	Number of subjects	Number of asthma outcomes (%)	Number of children with a parental history of asthma	Presence or absence of a parental history of asthma	Subgroup-specific hazard ratio	95% confidence interval
Single-exposure effect (with an interaction term with a parental history of asthma)						
$PM_{2.5}$	5,480	1,085 (19.8)	2,088	Presence	1.16	0.53–2.57
				Absence	1.60	1.04–2.47
Water damage/home dampness	4,760	1,012 (21.3)	1,762	Presence	1.09	0.88–1.34
				Absence	1.24	1.03–1.50
Dogs at home	5,707	1,105 (19.4)	2,157	Presence	0.77	0.61–0.98
				Absence	0.84	0.69–1.02
Cats at home	5,719	1,115 (19.5)	2,153	Presence	0.90	0.72–1.12
				Absence	0.96	0.79–1.16
Dust mite (Der f 1 or Der p 1) allergen in the child bed or bedroom floor	1,801	536 (29.8)	792	Presence	1.20	0.81–1.72
				Absence	1.20	0.73–1.97

Cox proportional hazards models were used to evaluate the single-exposure effects of $PM_{2.5}$ and indoor home exposures by including each of them in the model one at a time. Child's sex, race, ethnicity, parental history of asthma, maternal education, maternal smoking during pregnancy, % low-income neighborhood (Z score), and % Black, non-Hispanic neighborhood (Z score) were adjusted as covariates. Cohort-specific effects were implemented as fixed-effects. An interaction term between each exposure and the cohort status (high-risk vs. general-risk) defined by parental history of asthma or allergy was added to the models. $PM_{2.5}$ was scaled to reflect the change from the 10th to 90th percentile of the entire cohort (6.74 $\mu g/m^3$). We calculated the linear combination of regression coefficients of each exposure among those with and without a parental history of asthma.

Abbreviation: N=number, $PM_{2.5}$=particulate matter with an aerodynamic diameter ≤2.5 μm

eTable 7. Summary results of the sensitivity analysis for the outcome (single-exposure models)

Exposures	Number of subjects	Number of asthma outcomes (%)	Hazard ratio	95% confidence interval	p-value
Single-exposure effect (without adjustment for $PM_{2.5}$)					
The time to childhood asthma diagnosis between age 1 month to 12 years					
$PM_{2.5}$	5,480	1,521 (27.8)	1.17	0.85–1.61	0.33
Water damage/home dampness	4,760	1,363 (28.6)	1.09	0.97–1.23	0.14
Dogs at home	5,707	1,531 (26.8)	0.88	0.77–0.99	0.04
Cats at home	5,719	1,547 (27.1)	0.96	0.85–1.09	0.53
Dust mite (Der f 1 or Der p 1) allergen in the child bed or bedroom floor	1,801	682 (37.9)	1.18	0.91–1.51	0.21
The time to childhood asthma diagnosis which required any wheeze at age 3 or above					
$PM_{2.5}$	5,480	1,229 (22.4)	1.30	0.92–1.84	0.14
Water damage/home dampness	4,760	1,131 (23.8)	1.14	1.00–1.29	0.06
Dogs at home	5,707	1,205 (21.1)	0.86	0.74–0.99	0.03
Cats at home	5,719	1,212 (21.2)	0.95	0.83–1.09	0.49
Dust mite (Der f 1 or Der p 1) allergen in the child bed or bedroom floor	1,801	500 (27.8)	1.16	0.88–1.53	0.29

We applied Cox proportional hazards models to evaluate the single-exposure effects of $PM_{2.5}$ and indoor home exposures by including each of them in the model one at a time. Child's sex, race, ethnicity, parental history of asthma, maternal education, maternal smoking during pregnancy, % low-income neighborhood (Z score), and % Black, non-Hispanic neighborhood (Z score) were adjusted as covariates. Cohort-specific effects were implemented as fixed-effects. $PM_{2.5}$ was scaled to reflect the change from the 10th to 90th percentile of the entire cohort.

Abbreviation: N=number, $PM_{2.5}$=particulate matter with an aerodynamic diameter $\leq$2.5 μm

eTable 8. Summary results of the sensitivity analysis for the outcome (dual-exposure models)

Exposures	Number of subjects	Number of asthma outcomes (%)	Adjusted hazard ratio of an indoor exposure	95% confidence interval	p-value	Adjusted hazard ratio of $PM_{2.5}$	95% confidence interval	p-value
Independent exposure effect (with adjustment for $PM_{2.5}$)								
The time to childhood asthma diagnosis between age 1 month to 12 years								
Water damage/home dampness	4,067	1,208 (29.7)	1.07	0.94–1.21	0.33	1.15	0.77–1.70	0.50
Dogs at home	5,068	1,383 (27.3)	0.87	0.76–1.00	0.04	1.15	0.82–1.60	0.43
Cats at home	5,086	1,396 (27.4)	0.95	0.83–1.07	0.37	1.16	0.83–1.62	0.38
Dust mite (Der f 1 or Der p 1) allergen in the child bed or bedroom floor	1,534	586 (38.2)	1.16	0.90–1.50	0.25	1.56	0.85–2.84	0.15
The time to childhood asthma diagnosis which required any wheeze at age 3 or above								
Water damage/home dampness	4,067	1,055 (25.9)	1.10	0.97–1.26	0.15	1.32	0.88–1.97	0.18
Dogs at home	5,068	1,128 (22.3)	0.86	0.74–1.00	0.04	1.23	0.85–1.76	0.27
Cats at home	5,086	1,135 (22.3)	0.93	0.81–1.07	0.33	1.25	0.87–1.79	0.23
Dust mite (Der f 1 or Der p 1) allergen in the child bed or bedroom floor	1,534	455 (29.7)	1.13	0.85–1.49	0.39	1.23	0.62–2.46	0.56

As a sensitivity analysis, we assessed time to childhood asthma diagnosis which required any wheeze at age 3 or above. We used the Cox proportional hazards models to evaluate the independent exposure effects of $PM_{2.5}$ and each indoor home exposure by including each pair in the

model one at a time. Child's sex, race, ethnicity, parental history of asthma, maternal education, maternal smoking during pregnancy, % low-income neighborhood (Z score), and % Black, non-Hispanic neighborhood (Z score) were adjusted as covariates. Cohort-specific effects were implemented as fixed-effects. $PM_{2.5}$ was scaled to reflect the change from the 10th to 90th percentile of the entire cohort.

Abbreviation: N=number, $PM_{2.5}$=particulate matter with an aerodynamic diameter ≤2.5 μm
